# Early Policy Impact on Preventive Health: Regression Discontinuity Study of Singapore Healthier SG

**DOI:** 10.1093/geroni/igaf122.3749

**Published:** 2025-12-31

**Authors:** Seoyeon Ahn, Alec Morton, Zhi Zhen Lim, Shumian Yeo, Cynthia Chen, Linda Wei Lin Tan, Xueling Sim, Ian Yi Han Ang

**Affiliations:** Duke-NUS Medical School/National University of Singapore, Singapore, Singapore; National University of Singapore, Singapore, Singapore; National University of Singapore, Singapore, Singapore; National University of Singapore, Singapore, Singapore; National University of Singapore, Singapore, Singapore; National University of Singapore, Singapore, Singapore; National University of Singapore, Singapore, Singapore; National University of Singapore, Singapore, Singapore

## Abstract

**Background:**

Singapore’s Healthier SG program, launched in 2023, promotes voluntary primary care enrollment and preventive health screenings. Initially targeting adults aged 60 and above, eligibility expanded to those aged 40 and above by January 2024. This study evaluates the causal effect of program enrollment on screening uptake using regression discontinuity design centered at the age-40 eligibility threshold.

**Methods:**

We applied fuzzy regression discontinuity design using the age-40 cutoff to estimate local average treatment effects of Healthier SG enrollment on screening behavior. The analytic sample included 522 adults aged 37 to 42 from the Singapore Population Health Studies surveyed from June 2024 to January 2025. We estimated age-based eligibility effects on enrollment (first stage) and used two-stage least squares to assess enrollment impact on screening uptake.

**Results:**

Turning 40 increased enrollment probability by 38.6 percentage points (p < 0.001). In the second stage, enrollment raised screening uptake by 33.5 percentage points (95% CI: 13.9–53.1; p < 0.001). Reduced-form estimates showed that eligibility increased screening rates by 12.9 percentage points (p < 0.01). While the policy increased population-level screening modestly, it induced substantial behavioral change among enrollees. Results remained robust across model specifications, bandwidths, and covariate adjustments.

**Discussion:**

Healthier SG enrollment substantially increases screening uptake among newly eligible adults at age 40. The age-40 threshold represents a critical juncture for health system engagement to improve aging trajectories. These findings offer robust causal evidence for life-course triggers in preventive health policy and highlight the value of early mid-life interventions.

